# Dysregulation of Cytoskeleton Remodeling Drives Invasive Leading Cells Detachment

**DOI:** 10.3390/cancers13225648

**Published:** 2021-11-11

**Authors:** Jei-Ming Peng, Wei-Yu Chen, Jai-Hong Cheng, Jia-Wun Luo, Hong-Tai Tzeng

**Affiliations:** 1 Institute for Translational Research in Biomedicine, Kaohsiung Chang Gung Memorial Hospital, Kaohsiung 83301, Taiwan; wychen624@cgmh.org.tw (W.-Y.C.); pular124@cgmh.org.tw (J.-W.L.); 2 Center for Shockwave Medicine and Tissue Engineering, Medical Research, Kaohsiung Chang Gung Memorial Hospital and Chang Gung University College of Medicine, Kaohsiung 83301, Taiwan; cjh1106@cgmh.org.tw; 3 Department of Leisure and Sports Management, Cheng Shiu University, Kaohsiung 83301, Taiwan

**Keywords:** invasive leading cells, TGF-β1, actin cytoskeleton remodeling, vimentin intermediate filament networks, cell detachment

## Abstract

**Simple Summary:**

Detachment of cancer cells is the first step in tumor metastasis and malignancy. Our results showed that the TGF-β1/vimentin/focal adhesion protein assembly axis was involved in the control of the dynamics of initial tumor detachment under adequate nutrition, based on the Boyden chamber and 3D in-gel spheroid analysis.

**Abstract:**

Detachment of cancer cells is the first step in tumor metastasis and malignancy. However, studies on the balance of initial tumor anchoring and detachment are limited. Herein, we revealed that the regulation of cytoskeleton proteins potentiates tumor detachment. The blockage of TGF-β1 using neutralizing antibodies induced cancer cell detachment in the Boyden chamber and 3D in-gel spheroid models. Moreover, treatment with latrunculin B, an actin polymerization inhibitor, enhanced cell dissociation by abolishing actin fibers, indicating that TGF-β1 mediates the formation of actin stress fibers, and is likely responsible for the dynamics of anchoring and detachment. Indeed, latrunculin B disrupted the formation of external TGF-β1-induced actin fibers and translocation of intracellular vinculin, a focal adhesion protein, resulting in the suppression of cell adhesion. Moreover, the silencing of vimentin substantially reduced cell adhesion and enhanced cell detachment, revealing that cell adhesion and focal adhesion protein translocation stimulated by TGF-β1 require vimentin. Using the 3D in-gel spheroid model, we found that latrunculin B suppressed the cell adhesion promoted by external TGF-β1, increasing the number of cells that penetrated the Matrigel and detached from the tumor spheres. Thus, cytoskeleton remodeling maintained the balance of cell anchoring and detachment, and the TGF-β1/vimentin/focal adhesion protein assembly axis was involved in the control dynamics of initial tumor detachment.

## 1. Introduction

Mechanotransduction, the conversion of mechanical stimuli into cellular signals, promotes cytoskeleton remodeling and regulates gene expression, which contributes to the cells responding appropriately to the environment. Cytoskeletal remodeling transduces external mechanical signals into internal responses [1,2]. Cells and the external environment are connected through the cytoskeleton, which transmits external signals to influence cell behavior, including cell protrusion, adhesion, invasion, and metastasis [3]. After the cytoskeleton receives external stimulation, it activates the adhesion protein (focal adhesion kinase, FAK), the migration proteins (small GTP binding protein: RhoA, Cdc42, and Rac1), and the proliferation pathway (Hippo Pathway, JAK/STAT, and PI3K-AKT pathways) [4]. However, studies on the balance of mechanical transduction in cancer treatment are scarce. Understanding the correlation between pathological mechanical forces and signaling pathways in metastatic cancer can provide different therapeutic strategies.

During the epithelial–mesenchymal transition (EMT), TGF-β activation causes cytoskeleton remodeling through autocrine and paracrine pathways, leading to invasion and metastasis [5]. When the ECM is highly sclerotized, it increases the mechanical resistance to cell stretching and promotes the activation of TGF-β. TGF-β inhibits the expression of claudins, occludins, and ZO1, and disrupts cell–cell contact. Activated TGF-β then increases the expression of the downstream EMT transcription factors snail, MRTF, and ZEB, and promotes their activity through the SMAD-dependent pathway [6,7,8]. In addition, TGF-β also stimulates the activation of Rho GTPases, p38MAPK, and ERK1/2 through the SMAD-independent pathway [9]. TGF regulates the actin cytoskeleton by activating the RhoA-LIMK2-cofilin-1 pathway [10] and increases actin polymerization in cancer cells. These regulatory mechanisms promote actin recombination, leading to the formation of lamellipodia and filopodia, further facilitating cell metastasis [11,12,13]. The success rate of immunotherapy is dependent on the ability of immune cells to invade cancer cells [14]. The mechanism of immunotherapy, which primarily involves binding cell membrane surface proteins (including PD-L1, PD-1, and CTLA4) to suppress the attack of immune cells, has been discussed in numerous articles. Mariathasan et al. [15] found that the use of TGF-β1-blocking antibodies significantly promoted the infiltration of CD3^+^ T cells. However, there are limited studies on actin cytoskeleton remodeling in response to immune cell invasion.

Cell adhesion or movement requires the direct interaction between actin and vimentin filaments, which is mediated by the tail domain of vimentin [16,17]. Vimentin controls FAK activity through the VAV2-Rac1 pathway in cancer cells [18]. Vimentin modulates migration and EMT by upregulating Axl or Slug and Ras [19]. Focal adhesion proteins have been extensively studied in terms of survival and metastasis [20,21]. FAK1 protects the immune system by driving the fibrotic and immunosuppressive microenvironment, thus leading to tumor resistance to immunotherapy [22]. The inhibition of myosin II activity disrupts cytoskeleton remodeling and enhances the efficacy of immune checkpoint inhibitors [23].

The upregulation of TGF-β-1-mediated vimentin plays a key role in EMT progression [24]. Activated vimentin drives EMT and cancer dissemination by orchestrating the focal adhesion complex and cytoskeleton remodeling [25]. The blockage of TGF-β and inhibition of focal adhesion and enhanced immunotherapy indicate the possibility that cytoskeleton remodeling is involved in immune tolerance. The goal of this study was to clarify whether the autocrine TGF-β-vimentin-focal adhesion assembly axis contributes to the balance of adhesion and detachment by modulating focal adhesion protein translocation, and to determine whether abolishing cytoskeleton remodeling disrupts this balance and potentiates cell detachment.

## 2. Results

### 2.1. Sufficient Nutrients Induced the Detachment of Invasive Leading Cells

**Figure 1:**
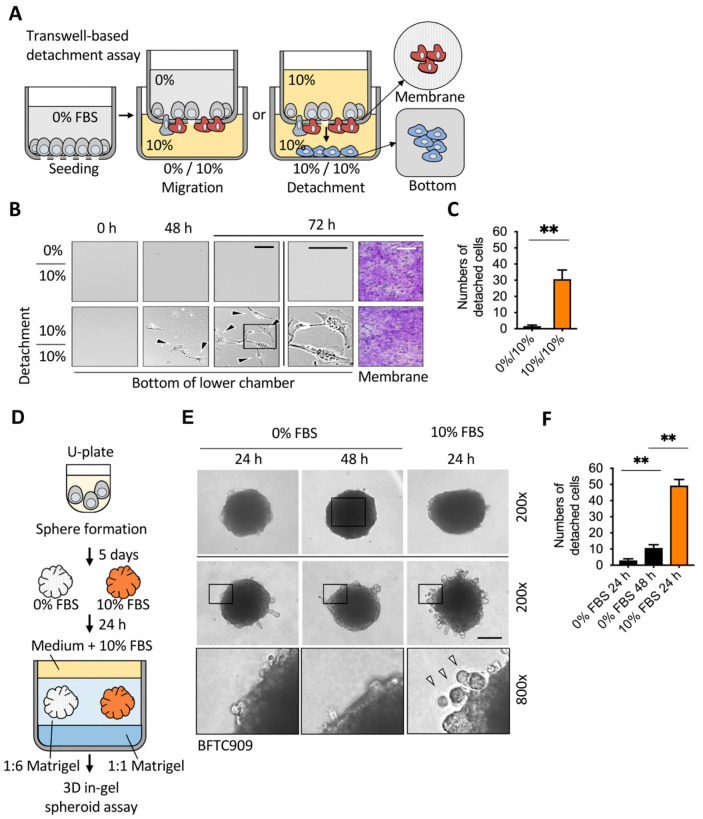
Detachment of invasive leading cells in the Boyden chamber and 3D in-gel spheroid detachment assay. (**A**) Schematic showing the procedure used for establishing a Boyden chamber detachment assay. (**B**) Representative images of the lower chamber demonstrated that starvation of cancer cells inhibited detachment in the leading cells. BFTC909 cells were seeded in the upper chamber overnight and the transwell assay was performed with or without serum in the upper chamber for 48 and 72 h. The membranes were stained by 1% crystal violet and the cell number from membranes or the lower chamber was determined (**B**,**C**, respectively). Scale bars: 100 μm. ** *p* < 0.01. Data are means ± s.d. (two-tailed *t*-test) from experiments with three replicates (*n* = 3). (**D**) Schematic showing the procedure used for establishing a 3D in-gel spheroid detachment assay. (**E**,**F**) In vitro 3D in-gel spheroid detachment assays demonstrated that the starvation of spheres led to reduced detachment in the leading cells. BFTC909 cells were plated in a 96-well U-plate for 5 days and the spheres were embedded in Matrigel for in-gel detachment assay. Detached cells from spheres were counted after 24 or 48 h. Black and white arrow: detached cells. Scale bars: 100 μm. ** *p* < 0.01. Data are means ± s.d. (one-way ANOVA with Dunnett’s multiple comparisons test) from experiments with three replicates (*n* = 3).

Tumor detachment is a part of tumor dissemination and metastasis [26]. To assess cell detachment, a Boyden chamber-based transwell assay was performed, as described for the migration assay. Traditionally, serum-free medium is added to the upper chamber, and 10% serum is added to the lower chamber to attract cells across the membrane. We found that if the upper medium is changed to contain 10% serum medium, cancer cells gain the ability to escape the membrane and reach the lower chamber bottom (Figure 1A,B). We examined the urothelial cancer cells, BFTC909, showing that when the upper chamber contained sufficient nutrients, it induced cell detachment in the lower chamber. There was no increase in the number of cells transferred under the membrane, but the number of detached cells at the bottom of the chamber increased significantly over time (Figure 1C). 

To further confirm whether low nutrients can also reduce cell detachment in 3D tumor spheres, we performed a 3D in-gel spheroid detachment assay (Figure 1D). First, cells were concentrated by low-speed centrifugation and cultured in a 96-well U-plate for 5–7 days to form tumor spheres. The spheres were embedded in the Matrigel for 24–48 h. The number of detached cells was calculated by integrating the two-dimensional section of invasion that lies in the perimembrane (Figure 1E,F). A supplement of BFTC909 cells with serum in the medium induced cell detachment, indicating that nutrients inside the tumor were not only required for growth but also involved in tumor dissemination. These data indicated that cell detachment levels were elevated in serum-supplied medium in a Boyden chamber and 3D in-gel spheroid detachment assay.

### 2.2. Blockage of TGF-β1 Enhanced Cell Detachment after Transmembrane Migration

**Figure 2:**
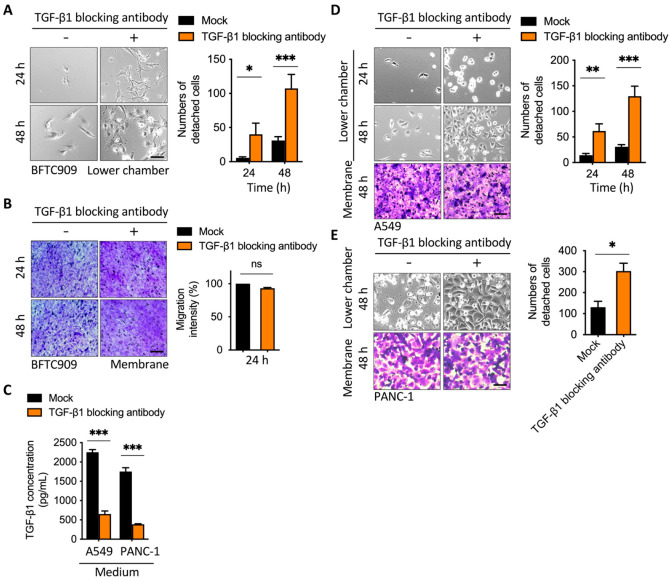
Blockage of TGF-β1 increased cell detachment after transmembrane migration. (**A**) Representative images of lower chamber and (**B**) transwell membrane demonstrated that the treatment of cancer cells with TGF-β1-blocking antibody increased detachment in the leading cells. BFTC909 cells were seeded in the upper chamber overnight and the transwell assay was performed with or without TGF-β1-blocking antibody in the lower chamber for 48 h. The membranes were stained by crystal violet and the cell number from membranes or the lower chamber was counted. Scale bars: 100 μm. * *p* < 0.05; *** *p* < 0.001. Data are means ± s.d. (two-way ANOVA with Tukey’s multiple comparisons test and two-tailed *t*-test, **A**,**B**, respectively) from experiments with three replicates (*n* = 3). ns, not significant. (**C**) ELISA assay showing treatment of TGF-β1-blocking antibody reduced TGF-β1 from the medium of the lower chamber. *** *p* < 0.001. Data are means ± s.d. (two-tailed *t*-test) from experiments with three replicates (*n* = 3). (**D**,**E**) Representative images of lower chamber and transwell membrane demonstrated that the treatment of A549 and PANC-1 cells with TGF-β1-blocking antibody increased detachment in the leading cells. Scale bars: 100 μm. (* *p* < 0.05; ** *p* < 0.01; *** *p* < 0.001). Data are means ± s.d. (two-way ANOVA with Tukey’s multiple comparisons test and two-tailed *t*-test, D and E, respectively) from experiments with three replicates (*n* = 3). Mock, mouse IgG1.

During metastasis and intravasation, cancer cells can dissociate from the tumor through the reduction in E-cadherin [26]. External TGF-β1 plays a role in tumor metastasis, but its role in cell detachment has not been studied [27]. Therefore, we wanted to understand whether TGF-β1 plays a regulatory role in cell detachment. To examine the effect of TGF-β1 on cell adhesion or detachment after transmembrane migration from the upper chamber with cancer cells, we first analyzed detachment levels in three types of cancer cells, which are known to cause tumor metastasis. According to the experimental design shown in Figure 1A, a transwell assay was performed and exchanged with serum-containing medium in the upper chamber after migration. BFTC909 in the lower chamber was treated with or without TGF-β1-blocking antibody, which is known to bind and reduce the effect of TGF-β1, and mouse IgG1 antibody was used in the control group. Interestingly, the decrease in TGF-β1 led to an increase in cell detachment after transwell migration (Figure 2A). We determined the cell number in the lower side of the membrane and found that treatment with TGF-β1-blocking antibody did not further affect cell migration (Figure 2B and Figure S1A,B). The effect of TGF-β-blocking antibody on cell migration in the 2D wound healing assay was also examined. Our results revealed that treatment with anti-TGF-β-blocking antibody did not affect cell migration when the seeding cell number was 5 × 10^3^ cells per well. However, the same treatment increased migration approximately 1.15-fold when the seeding cell number was 1 × 10^4^ cells per well, indicating that TGF-β inhibition induced cell migration under a higher cell density in the wound healing assay (Figure S2A,B). Ungefroren et al. performed similar experiments and found that the antibody-mediated neutralization of autocrine TGF-β promoted cell motility in the Boyden chamber assay within 24 h [28]. These results suggested that the inhibition of autocrine TGF-β might increase migration in the early time of the Boyden Chamber assay and then induce cell detachment after cell migration to the lower side of the membrane.

TGF-β1 could cause the opposite effect in either EMT or cell death [29], whereas in our data, the disruption of the effect of TGF-β1 by a blocking antibody did not further increase migration levels but largely induced cell detachment, indicating that TGF-β1 was required for cell anchorage after transwell migration (Figure 2A,B). To confirm the effect of cell proliferation in cell detachment, the treatment of mitomycin C in the Boyden chamber was performed. Low doses of mitomycin C (0.15 and 0.31 µM) had not affected cell detachment, while high doses of mitomycin C caused cell death and reduced detached cells in the lower chamber (Supplementary Figure S3). The effect of TGF-β1-blocking antibody was determined by the TGF-β1 ELISA assay (Figure 2C). We also demonstrated that the blockage of TGF-β1 enhanced cell detachment in A549 (lung cancer cell) and PANC-1 (pancreatic cancer cell) and significantly induced cell detachment (Figure 2D,E). Thus, our data demonstrate that TGF-β1 is involved in cell anchorage after transwell migration in several types of cancer cells, suggesting that there is a feedback regulation of EMT and cytoskeleton remodeling in the balance of cell adhesion and detachment after migration.

### 2.3. Disruption of Actin Cytoskeleton Remodeling Led to Reduction in TGF-β1-Induced Transmembrane Anchoring

**Figure 3:**
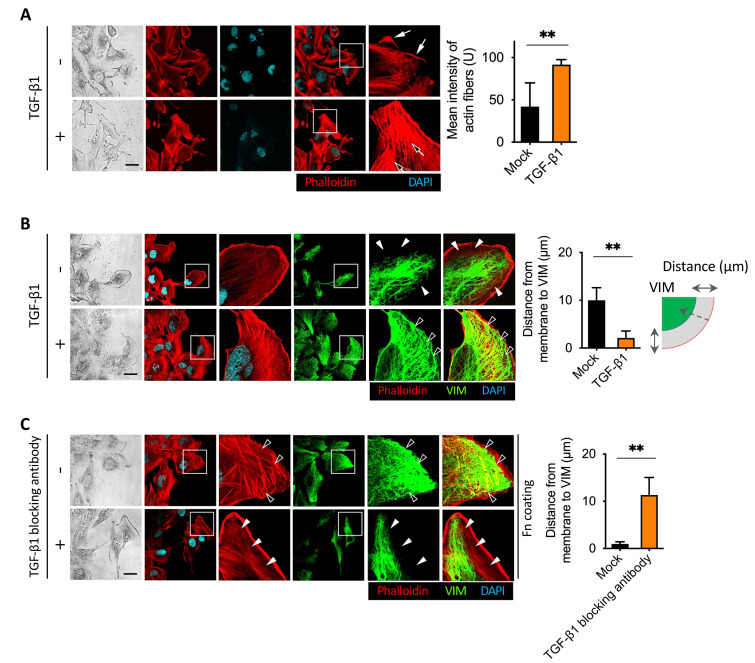
TGF-β1 induced vimentin intermediate filament networks and actin stress fibers formation. (**A**) Representative immunofluorescence assay of TGF-β1-induced actin stress fibers formation in the leading cells. BFTC909 cells were seeded in a wound healing chamber overnight, and the migration was performed for 24 h, followed by treatment with or without TGF-β1 ligands for another 24 h. The cells were then fixed and stained with phalloidin, and the intensity of the stress fibers was quantified and plotted. Black arrow: actin stress fibers. White arrow: lamellipodia. Scale bars: 10 μm. ** *p* < 0.01. Data are means ± s.d. (two-tailed *t*-test) from experiments with three replicates (*n* = 8 fields for each experiment). (**B**) Representative images showing that TGF-β1 promoted vimentin intermediate filament networks and actin stress fibers formation in BFTC909 cells. ** *p* < 0.01. Data are means ± s.d. (two-tailed *t*-test) from experiments with three replicates (*n* = 8 fields for each experiment). (**C**) Representative images showing that TGF-β1-blocking antibody reduced vimentin intermediate filament networks and actin stress fibers formation in BFTC909 cells. Arrowheads: the space between the intracellular membrane and vimentin. Scale bars: 10 μm. ** *p* < 0.01. Data are means ± s.d. (two-tailed *t*-test) from experiments with three replicates (*n* = 8 fields for each experiment).

Actin cytoskeleton remodeling is involved in the EMT and MET transition [30]. TGF-β1 promotes the formation of stress fibers and intracellular focal adhesion [31]. We next wanted to know whether the disruption of actin stress fibers is correlated with cell detachment. First, we examined whether TGF-β1 promoted the formation of stress fibers in the cells we used. We conducted a wound healing assay using BFTC909. The migrating cells in the leading edge of the wound healing assay showed stark actin stress fibers, vimentin intermediate filaments, and vinculin accumulation; therefore, it is beneficial to observe the effects of inhibitors on structural proteins. After treatment with TGF-β1 for 24 h, it was clearly detected through an immunofluorescence assay that TGF-β1 promoted the formation of actin stress fibers (Figure 3A). As TGF-β1 can promote vimentin intermediate filament networks, we found that vimentin filaments extended to the cell membrane after TGF-β1 treatment (Figure 3B). Mariathasan et al. demonstrated that using anti-TGF-β1 antibody can block TGF-β1-induced signaling [15]. We further determined whether TGF-β1 blocking affected vimentin intermediate filament networks. First, we coated glass slides with fibronectin to induce actin stress fibers. We then compared actin stress fibers and vimentin intermediate filaments with or without using the anti-TGF-β1 antibody. We found that treatment with an anti-TGF-β1 antibody inhibited actin stress fibers and vimentin intermediate filament networks (Figure 3C). These results suggest that external TGF-β1 increased actin stress fibers and vimentin intermediate filament networks of invasive leading cells after migration. This result was consistent with the result that the blockage of autocrine TGF-β1 enhanced cell detachment after transmembrane migration (Figure 2). The blockage of TGF-β1 inhibited actin stress fibers and vimentin intermediate filament networks and promoted cell detachment.

**Figure 4:**
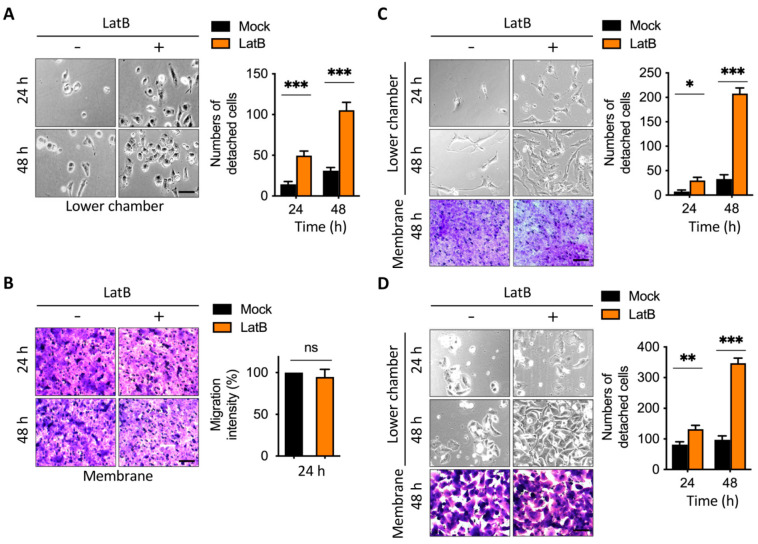
Latrunculin B disrupted autocrine TGF-β1-induced transmembrane anchoring. (**A**,**B**) Images from the representative 2.5D detachment assay; A549 cells were seeded in the upper chamber overnight and the transwell assay was performed with or without serum in the upper chamber for 24 and 48 h. The membranes were stained by crystal violet and the cell number from the membranes or lower chamber was measured. Scale bars: 100 μm. *, *p* < 0.05; ***, *p* < 0.001. Data are means ± s.d. (two-way ANOVA with Tukey’s multiple comparisons test and two-tailed *t*-test, A and B, respectively) from experiments with three replicates (*n* = 3). (**C**,**D**) Addition of latrunculin B enhanced the leading cells detachment in BFTC909 and PANC-1. Scale bars: 100 μm. *, *p* < 0.05; **, *p* < 0.01; ***, *p* < 0.001. Data are means ± s.d. (two-way ANOVA with Tukey’s multiple comparisons test) from experiments with three replicates (*n* = 3).

Additionally, we determined whether direct interference with actin stress fibers also affects the transmembrane anchoring caused by external TGF-β1. According to our previous data [30], latrunculin B disrupts actin cytoskeleton remodeling and induces cell migration through the activation of snail under high cell density and nutrient deficiency. Therefore, we tested whether the shortening of actin stress fibers caused by latrunculin B affected cell detachment. To test whether the cell anchorage effect of TGF-β1 is dependent on actin cytoskeleton remodeling, we incubated cells with latrunculin B, an actin filament modulator, which functions in actin depolymerization [32]. Using the transwell assay as described (Figure 2), the medium in the lower chamber was added with or without latrunculin B, which abolished the effect of TGF-β1-induced transmembrane anchoring (Figure 4A). To further confirm the migration of cells in the membrane of the upper chamber, we stained the membrane with crystal violet. There were no significant differences between the cell number of latrunculin B-treated groups and the control group in the transmembrane (Figure 4B), indicating that latrunculin B promoted cell detachment from the transmembrane. To further validate our findings in other human cancer cell lines, we treated the medium of the lower chamber with latrunculin B, which also enhanced cell detachment in BFTC909 and PANC-1 cells (Figure 4C,D). Together, these results suggest that the addition of the actin filament modulator, latrunculin B, suppressed actin cytoskeleton remodeling and enhanced cell detachment in a transwell migration model.

### 2.4. Latrunculin B Disrupted TGF-β1-Mediated Actin Stress Fibers and Vimentin Intermediate Filament Networks 

**Figure 5:**
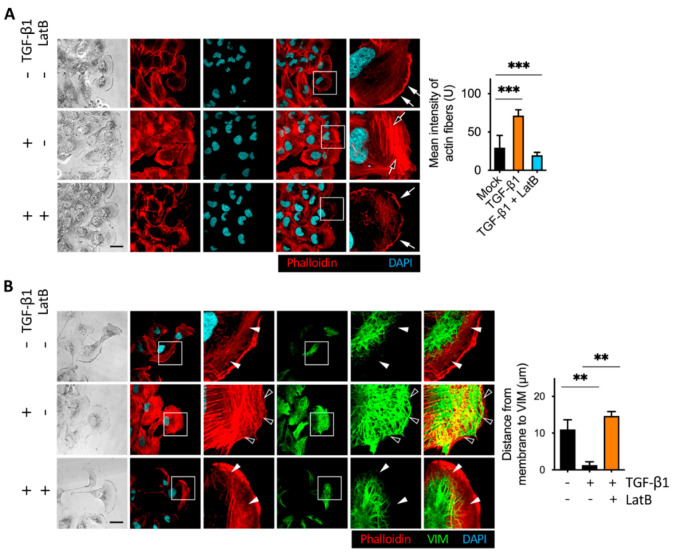
Latrunculin B disrupted TGF-β1-mediated actin stress fibers formation and vimentin intermediate filament networks. (**A**) Images of representative immunofluorescence results revealed that the addition of latrunculin B disrupted TGF-β1-induced actin stress fibers formation. BFTC909 cells were seeded in the wound healing chamber overnight, followed by migration with or without treatment of TGF-β1 ligands or latrunculin B for 24 h. Black arrow: actin stress fibers. White arrow: lamellipodia. Scale bars: 10 μm. *** *p* < 0.001. Data are means ± s.d. (one-way ANOVA with Dunnett’s multiple comparisons test) from experiments with three replicates (*n* = 3). (**B**) Treatment of latrunculin B repressed vimentin intermediate filament networks in BFTC909. Migration was performed with or without treatment of TGF-β1 ligands or latrunculin B for 24 h. Arrowheads: the space between the intracellular membrane and vimentin. Scale bars: 100 μm. ** *p* < 0.01. Data are means ± s.d. (one-way ANOVA with Dunnett’s multiple comparisons test) from experiments with three replicates (*n* = 3). LatB, latrunculin B.

To determine whether the addition of latrunculin B induced cell detachment through TGF-β1-induced actin stress fibers formation and vimentin intermediate filament networks, we performed immunofluorescence analysis followed by a wound healing assay. First, we examined the role of TGF-β1 in actin cytoskeleton remodeling. The expression of stress fibers was detected by immunofluorescence staining with phalloidin. We observed that fewer actin stress fibers were formed in the cytoplasm after 24 h of migration, while TGF-β1 treatment significantly enhanced the bundles of stress fibers in the invasive leading cells (Figure 5A). We found that latrunculin B abolished actin stress fibers, which are known to be responsible for focal adhesion protein accumulation and membrane binding (Figure 5A). Thus, treatment with latrunculin B disrupted the actin stress fibers formation. Jiu et al. [33] revealed that vimentin architects actin cytoskeleton remodeling. To examine the effect of latrunculin B on TGF-β1-mediated vimentin intermediate filaments and stress fibers formation in the invasive leading cells after migration, we performed a wound healing assay followed by immunofluorescence staining with vimentin and phalloidin, and eight field images were analyzed (Figure 5B). Treatment with latrunculin B downregulated the effect of TGF-β1 and reduced vimentin intermediate filament networks and actin bundle expression, which may grant cells the ability to detach, as shown in Figure 2.

**Figure 6:**
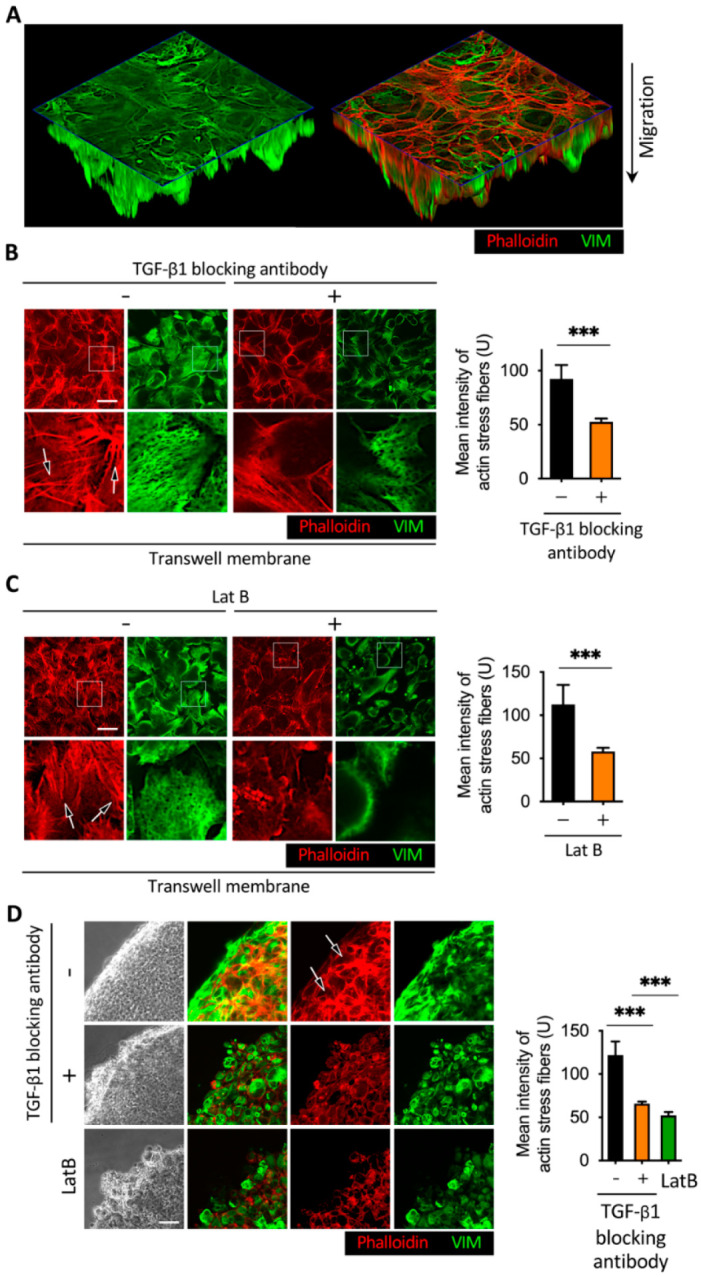
Effect of TGF-β1 neutralizing antibody and latrunculin B in the Boyden chamber and 3D sphere assay. (**A**) Immunofluorescence images of the Boyden chamber assay. (**B**,**C**) Images of representative immunofluorescence results revealed that the addition of TGF-β1-blocking antibody or latrunculin B reduced actin stress fibers and vimentin intermediate filament networks. BFTC909 cells were seeded in the Boyden chamber overnight, followed by migration for 48 h. A detachment assay was performed with or without treatment of TGF-β1 ligands or latrunculin B for another 48 h. Scale bars: 10 μm. *** *p* < 0.001. Data are means ± s.d. (two-tailed *t* test) from experiments with three replicates (*n* = 3). (**D**) Treatment of TGF-β1-blocking antibody or latrunculin B repressed actin stress fibers and vimentin intermediate filament networks in BFTC909. A three-dimensional sphere assay was performed with or without treatment of TGF-β1-blocking antibody or latrunculin B for 48 h. Black arrow: actin stress fibers. Scale bars: 10 μm. *** *p* < 0.001. Data are means ± s.d. (one-way ANOVA with Dunnett’s multiple comparisons test) from experiments with three replicates (*n* = 3). LatB, latrunculin B.

The effect of anti-TGF-β1 antibody and latrunculin B on structural proteins in the 2D wound healing assay may represent different phenomena in the Boyden chamber and in-gel spheroid assays, versus in the 2D wound healing assay. Therefore, we performed immunofluorescence staining in the Boyden chamber and 3D sphere assays to detect actin stress fibers, vimentin intermediate filaments, and vinculin. In the Boyden chamber assay, the anti-TGF-β1-blocking antibody or latrunculin B was added to the lower membrane for 48 h (Figure 6A–C). For the 3D sphere assay, the spheres were cultured in culture medium instead of Matrigel to perform immunofluorescence staining (Figure 6D). We detected actin stress fibers, vimentin intermediate filaments, and vinculin accumulation in the lower side membrane of the Boyden chamber (Figure 6B,C). These actin stress fibers and vimentin intermediate filament networks were also detectable in the 3D sphere assay (Figure 6D). Forty-eight hours after treatment with anti-TGF-β antibody and latrunculin B, the results of cytoskeleton protein staining in the Boyden chamber and 3D sphere assays were similar to those in the 2D wound healing assay. Treatment with anti-TGF-β antibody or latrunculin B inhibited actin stress fiber formation and vimentin intermediate filament networks. Vinculin was only detectable in the Boyden chamber assay but was not visible in the 3D sphere assay.

### 2.5. Silencing of Vimentin Led to a Reduction in Membrane Anchoring in the Invasive Leading Cells

**Figure 7:**
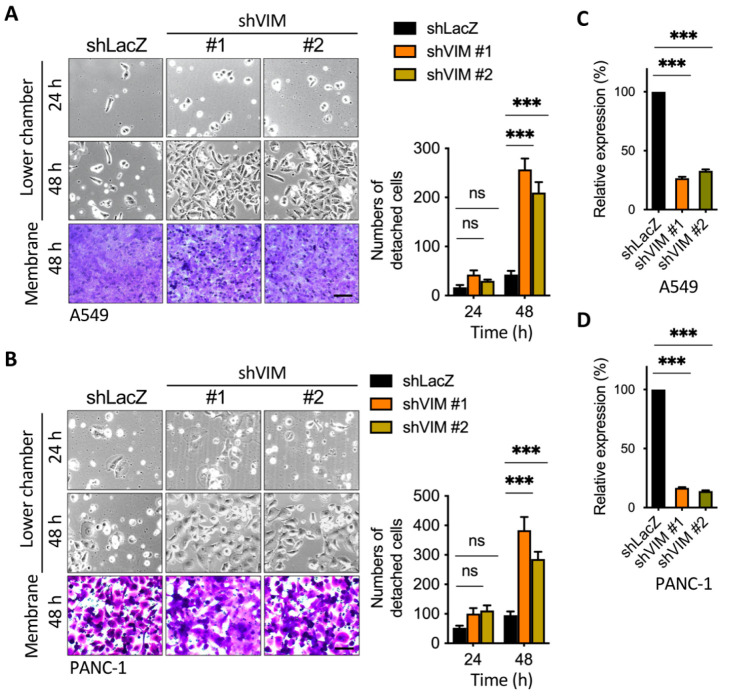
Silencing of vimentin induced cell detachment. (**A**,**B**) Images from the representative transwell assay; silencing of vimentin increased the leading cells detachment. A549 and PANC-1 cells were silenced with shLacZ or shVIM and seeded in the upper chamber overnight, and the transwell assay was performed for 24 and 48 h. The membranes were stained by crystal violet and the cell number from membranes or the lower chamber was counted. Scale bars: 100 μm. *** *p* < 0.001. Data are means ± s.d. (two-way ANOVA with Tukey’s multiple comparisons test) from experiments with three replicates (*n* = 3). (**C**) Relative expressions of vimentin mRNA in BFTC909-shLacZ, -shVIM#1, and shVIM#2 were determined by the real-time qPCR assay. (**D**) Relative expressions of vimentin mRNA in PANC-1-shLacZ, -shVIM#1, and shVIM#2 were determined by the real-time qPCR assay. *** *p* < 0.001. Data are means ± s.d. (one-way ANOVA with Dunnett’s multiple comparisons test) from experiments with three replicates (*n* = 3). VIM, vimentin.

We further examined the effect of vimentin on cancer cell detachment by generating small short hairpin RNAs (shRNAs) targeting vimentin in A549 and PANC-1 cells (Figure 7A,B). We then performed a transwell assay to determine the effect of shVimentin on cell detachment. Most notably, the knockdown of endogenous vimentin by shRNAs increased cell detachment in comparison to the control shRNA, and two cell lines revealed similar results (Figure 7A,B). The silencing efficiency was confirmed by real-time qPCR in both A549 and PANC-1 cells (Figure 7C,D). These data imply that both vimentin and actin cytoskeleton remodeling are required to mediate anchorage or detachment after migration.

### 2.6. Latrunculin B-Induced Focal Adhesion Proteins Translocation and Cell Detachment from Tumor Spheres

**Figure 8:**
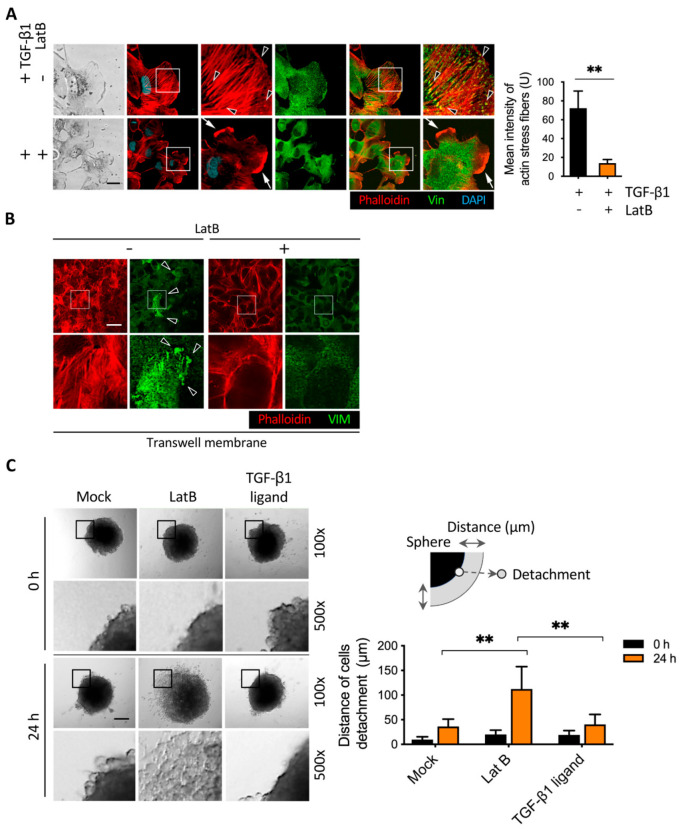
Latrunculin B induced focal adhesion complex translocation and cells spatial detachment. (**A**) Images of representative immunofluorescence results showed that adding latrunculin B induced vinculin translocation. BFTC909 cells were seeded in a wound healing chamber overnight, followed by migration with or without treatment of TGF-β1 ligands or latrunculin B for 24 h. Black arrow: actin stress fibers. White arrow: lamellipodia. (**B**) Treatment of latrunculin B induced vinculin translocation in the Boyden chamber assay. The cells were fixed and stained with anti-vinculin antibody and phalloidin. Arrowheads: vinculin accumulation. Scale bars: 10 μm. ** *p* < 0.01. Data are means ± s.d. (two-tailed *t*-test) from experiments with three replicates (*n* = 3). (**C**) Treatment of latrunculin B increased cell detachment in a 3D in-gel spheroid assay. The spheres were embedded in the materiel with 10% FBS for 24 h. Scale bars: 100 μm. ** *p* < 0.01. Data are means ± s.d. (two-way ANOVA with Tukey’s multiple comparisons test) from experiments with three replicates (*n* = 3). LatB, latrunculin B.

As shown in Figure 3A, latrunculin B promoted cell detachment after transwell migration. According to a review report by Hinz et al. [5], TGF-β1 induced the formation of actin stress fibers and resulted in intracellular focal adhesion assembly. During migration, focal adhesion is known to translocate to the perimembrane of cells [34], and the larger size of focal adhesion formed in the intracellular membrane was responsible for cell anchorage. However, their role in controlling focal adhesion complex translocation to balance cell anchoring and detachment has been less reported. To explore the correlation between focal adhesion proteins and cell detachment in our model, we examined the effect of latrunculin B on the TGF-β1-mediated effect in vinculin, a representative focal adhesion protein, translocation to the perimembrane in migrating cells. We first determined vinculin expression in the perimembrane in the invasive leading cells, while the addition of TGF-β1 promoted vinculin accumulation with a high expression of actin stress fibers, suggesting that the TGF-β1-actin stress fibers-vinculin axis was utilized as a feedback mechanism for the regulation of cell migration (Figure 8A). We added latrunculin B to cells for which a wound healing assay was performed with or without TGF-β1 treatment. Immunofluorescence staining showed that treatment with TGF-β1 enhanced intracellular vinculin formation, and co-treatment with latrunculin B significantly decreased actin bundle formation in leading cells (Figure 8A). Abundant lamellipodia were formed after the addition of latrunculin B, suggesting that latrunculin B disrupts TGF-β1-induced intracellular vinculin and adhesion (Figure 8A). We also detected the effect of latrunculin B on actin stress fibers and vinculin accumulation in the lower side membrane of the Boyden chamber (Figure 8B). Treatment with latrunculin B diminished vinculin accumulation at focal adhesions and reduced intracellular focal adhesion strength, which may provide an explanation in Figure 4A that latrunculin B induced cell detachment by disrupting TGF-β1-mediated anchoring. 

We further demonstrated that latrunculin B induced cell detachment in a 3D in-gel spheroid detachment model. Most notably, the 3D spheres that expressed cell detachment were enhanced by the addition of latrunculin B in comparison to the control, indicating that latrunculin B can disrupt cell adhesion status under both Boyden chamber and 3D growth conditions (Figure 8C). Treatment with latrunculin B obviously stimulated cell detachment from tumor spheres, and the result was not found in the TGF-β1-treated spheres. Cell–cell dissociation and migration occur in the in-gel spheroid assay. In our 3D in-gel spheroid detachment assay, the spheres were embedded in the Matrigel with serum-supplied medium. After 24 h, cell–cell dissociation occurred, but cell migration was not obvious under this condition (Figure 8C). It revealed a lack of ligands in the Matrigel for integrins expressed in our tested cells. Moreover, LatB treatment did not increase cell migration in our 2D wound healing assay (Figure S2). Therefore, the enhancement of cell detachment after the addition of LatB should be mainly due to cell–cell dissociation rather than migration.

Our results revealed that serum application was required for cell dissociation from the membrane, whereas the number of detached cells without extra treatment (TGF-β antibody treatment) was low. This indicated that serum was required for cell detachment, while additional stimuli (disruption of cytoskeleton remodeling by treatment with TGF-β-blocking antibody and LatB) promoted the effect of cell dissociation. These results indicate that adequate nutrients are necessary for cell detachment, and extra stimuli improve the effect of cell dissociation. Collectively, these findings indicate that the effect of external TGF-β1 on adhesion was elevated in leading cells after migration, and this is likely to be impeded by treatment with a TGF-β1-blocking antibody, latrunculin B, and the silencing of vimentin, which were found to enhance cell detachment. Latrunculin B impedes TGF-β1-mediated actin cytoskeleton remodeling and vimentin intermediate filament networks, resulting in cell detachment in invasive leading cells.

## 3. Discussion

Initial cancer cell detachment occurs during intravasation, extravasation, and metastasis during tumor progression, as well as metastasis to distal organs [26]. As there have been few studies on balancing cancer cell anchoring and detachment, it is important to understand the mechanism of invasive leading cell detachment during tumor progression. Our study found that cytoskeletal protein remodeling mediated by TGF-β1 and vimentin affected the balance of cell adhesion and dissociation in human colorectal, lung, and pancreatic cancer cell lines (Figure 2, Figure 5, Figure 6 and Figure 7). Using an anti-TGF-β1-blocking antibody and an actin polymerization inhibitor, we confirmed that interfering with cytoskeleton remodeling leads to cancer cell detachment (Figure 3 and Figure 4). Furthermore, using the 3D detachment model, we found that interfering with cytoskeleton remodeling significantly promoted the invasive leading cell detachment of tumor spheres (Figure 8). These results may provide a reference for therapeutic strategies for tumors affected by relevant mechanisms.

TGF-β1 is a key factor in the regulation of tumor EMT and is related to anoikis. Anoikis is a process that causes apoptosis after cancer cells dissociate from the tumor. Malignant tumor cells are often resistant to anoikis [35]. Several proteins involved in TGF-β1 signaling have been shown to regulate anoikis and EMT, including DEAR1, which is encoded by a tumor suppressor gene that is frequently absent in patients with advanced breast cancer. DEAR1 promotes SMAD3 degradation by directly binding SMAD3 and inhibiting SMAD3 downstream target genes, including Snail and Slug [36]. DEAR1 deficiency proved to be necessary for TGF-β-induced EMT and anoikis resistance. In our study, we found that treatment with an anti-TGF-β1-blocking antibody interfered with TGF-β1-regulated cell adhesion and promoted cell detachment. However, it is speculated that when cells are detached from the ECM, anoikis may be increased via DEAR1, resulting in cell death. For malignant cancer cells that survive detachment, DEAR1 might be deficient in function and expression while increasing anoikis resistance and promoting cancer cell survival; nevertheless, this hypothesis requires further experimental evidence.

In addition to developing resistance to anoikis-dependent pathways, tumors develop anoikis-independent survival strategies. Autophagy is an important mechanism that regulates cell survival or death [37]. Autophagy is a process to break down intracellular components, such as lipids, proteins, or organelles, to promote survival in the absence of nutrients in the cell [38]. Using various cancer cell lines, Debnath et al. showed that autophagy is an effective strategy for ECM cell dissociation. In tissue culture cells, separation from the ECM is sufficient to induce autophagy [39]. Later studies identified a signaling mechanism by which the endoplasmic reticulum kinase PERK (also known as eIF2αK3) induces autophagy during ECM dissociation [40]. Upon cell detachment from the ECM, PERK induces the activation of AMP-activated protein kinase and tuberous sclerosis complex 2 and inhibits the mTORC1 complex [41]. In addition, during ECM detachment, autophagy can be driven by the IκB kinase complex, which regulates cell viability [42].

When cancer cells break away from the tumor, they alter the metabolic capacity and limit the nutrient-utilizing ability of cells. These changes may also contribute to cell death. Zhu et al. demonstrated that the loss of ECM attachment ability is directly related to the influence of cell metabolism [43]. After cell detachment, the PI3K-Akt signaling pathway is downregulated, leading to decreased glucose uptake. At this point, glucose uptake can be restored through the ERBB2-mediated regulatory signaling process to produce ATP. Additionally, it has been shown that the addition of antioxidants to detached cells compensates for the loss of glucose uptake and promotes ATP production [43,44].

ERBB2 can also inhibit PDK4 via ERK to promote ATP production in detached cells [45]. DeNicola et al. found that antioxidant enzymes are mainly produced by the transcription factor NFE2-related factor 2 (NRF2). NRF2 can promote the survival of detached cancer cells by affecting oncogenes such as RAS, RAF, and MYC [46]. As TGF-β1 is associated with vimentin and PERK, PI3K/AKT, and ERBB2-regulated functions, interference with TGF-β1- and vimentin-regulated signaling may affect the functions of these proteins. In our study, we found that blocking TGF-β1 and vimentin deletion induced cancer cells to detach and survive, suggesting that PERK, PI3K/AKT, and ERBB2 may also be involved in the survival of detached cells through increasing the anoikis-independent process.

TGF-β signaling can control the differentiation and behavior of cancer cells by promoting their elongation and enhancing their motility, thereby allowing them to migrate and invade the ECM. TGF-β promotes epithelial cell plasticity and EMT mainly by activating TβRI and the SMAD3/4-mediated transcription of major EMT transcription factors such as Snail1, Slug, ZEB1, and ZEB2 [47,48]. However, in our study, we found that cytoskeleton remodeling was required upon detachment of invasive leading cancer cells from the tumor. Cell separation was achieved by reducing the intensity of the focal adhesion protein assembly mediated by TGF-β1 and vimentin. Our study showed that dynamic cytoskeleton remodeling exists in TGF-β-induced anchoring and metastasis. TGF-β signaling mediated tumor invasiveness and enhanced TGF-β signaling-induced cancer cell anchoring, whereas weakened TGF-β signaling was required for detachment. 

In recent years, immunotherapy has attracted much attention, and it has been shown that the inhibition of TGF-β signaling can reduce tumor growth and enhance the infiltration of immune cells [15,49,50,51,52]. According to the results of our study, the inhibition of TGF-β signaling significantly reduced actin stress fibers formation and vimentin intermediate filament networks (Figure 3C and Figure 6B,D), suggesting that the effect of enhanced immune cell infiltration may be achieved by weakening the cytoskeleton structure of the tumor.

Recent studies have shown that TGF-β and vimentin play a role in immune suppression in many human cancers and are associated with tumor dissemination, metastasis, and poor prognosis [29]. This immune suppression may be related to the tumor immune microenvironment regulated by TGF-β, indicating the importance of TGF-β as a tumor marker and possibly as a target for inhibiting the infiltration of immune cells and their toxicity to cancer cells [53]. Our results show that autocrine TGF-β1- and vimentin-mediated cytoskeleton remodeling affects the adhesion and detachment of human cancer cells. We further found that the use of the anti-TGF-β1-blocking antibody interfered with cytoskeleton remodeling, leading to cancer cell detachment. Anti-TGF-β1 plays a positive role in human tumor environments; it may promote the vulnerability of tumor cells to the attack by immune cells. It is speculated that the infiltration of immune cells may be enhanced by weakening the cytoskeleton structure of the tumor. Our findings may provide an explanation for the outcome of immunotherapy for patients, as well as a reference for therapeutic strategies for tumors affected by relevant mechanisms. 

Nutrient deficiency is an environmental stressor during tumor growth. Our previous studies have revealed that, in large tumors with the deletion of tumor suppressor genes, nutrient deficiency could lead to tumor metastasis and malignancy [30]. Therefore, environmental nutrient deficiency sometimes plays a role in promoting the progression of cancer cells, which has been widely used to study autophagy and several genes involved in the cancer cell tolerance to nutrient deprivation [54,55]. In this study, we found that the disruption of cytoskeleton remodeling promoted the dissociation of tumor cells, and this process requires adequate environmental nutrients.

## 4. Materials and Methods

### 4.1. Cell Lines and Materials

Human cells, BFTC909 (renal pelvis and urothelial cancer, 60069), were obtained from the Bioresource Collection and Research Center (BCRC). A549 (lung cancer, CCL-185) and PANC-1 (pancreatic cancer, CRL-1469) cell lines were obtained from the American Type Culture Collection (ATCC). BFTC909, A549, PANC-1, and their shRNA silencing derivatives were cultured in DMEM medium (Thermo Fisher Scientific, Waltham, MA, USA) containing 10% (*volume*/*volume*; *v*/*v*) fetal bovine serum (FBS), sodium bicarbonate, and 1% (*v*/*v*) penicillin/streptomycin at 37 °C in an incubator with a humidified atmosphere of 5% CO_2_. Cells were treated with actin cytoskeleton modulators, latrunculin B (Sigma-Aldrich, Missouri, MO, USA). In the 3D sphere in-gel detachment assay, the Matrigel (354234, Corning, Bedford, MA, USA) was diluted in DMEM and used at concentrations of 10 μg/mL and 20 μg/mL. The following antibodies were used in this study: monoclonal mouse anti-vimentin (V6389; Sigma; 1:1000 for Western blot, WB; 1:400 for immunofluorescence staining), monoclonal mouse anti-vinculin (FAK100; Sigma; 1:200 for IF), Alexa Fluor 594 conjugated phalloidin (A12381; Invitrogen; 1:200 for IF), and Alexa Fluor 488 goat anti-mouse (A11029; Invitrogen; 1:200 for immunofluorescence staining). For the transwell migration assay, a TGF-β1-blocking antibody (MA5-23795, Invitrogen, Waltham, MA, USA) was used.

### 4.2. shRNA, Lentivirus Infection, and Quantitative Real-Time PCR

To silence vimentin, shRNAs were selected and obtained from the National RNAi Core (Academic Sinica, Taiwan). The shRNA targets were shVimentin #1, GCTAACTACCAAGACACTATT; shVimentin #2, GCAGGATGAGATTCAGAATAT. Cancer cells were infected with shVimentin and control shRNA for 48 h prior to puromycin selection, and shRNA lentivirus at a multiplicity of infection (MOI) of 2 was used. For puromycin selection, 1 μg/mL was used for BFTC909, A549, and PANC-1 cells. Total RNA was extracted with TRIzol reagent (Thermo Fisher Scientific, Waltham, MA, USA), and cDNA was synthesized using SuperScript III reverse transcriptase (Thermo Fisher Scientific, Waltham, MA, USA). The silencing effect of shRNA was measured using real-time quantitative PCR (RT-qPCR) using the iQ SYBR Green supermen and an iCycler iQTM detection system (Bio-Rad, Laboratories, Hercules, CA, USA) according to the manufacturer’s instructions. The real-time PCR primers were as follows: vimentin forward, 5′-AGGCAAAGCAGGAGTCCACTGA-3′; vimentin reverse primer, 5′-ATCTGGCGTTCCAGGGACTCAT-3′. β-actin forward: 5′-CACCATTGGCAATGAGCGGTTC-3′; β-actin reverse primer: 5′-AGGTCTTTGCGGATGTCCACGT-3′. The 2^−^^∆∆Ct^ method was used to represent mRNA expression levels by normalizing to the housekeeping gene (actin).

### 4.3. Boyden Chamber Migration and Detachment Assay

Cell migration was assessed using a 6.5 mm transwell insert diameter with a pore size of 8 μm (Corning, Bedford, MA, USA). Cells were then seeded in the upper chambers with 300 μL of complete medium and left to settle overnight at 37 °C in an incubator with a humidified atmosphere of 5% CO_2_. The next day, the medium in the upper chamber was changed with or without serum (for migration or detachment assay, respectively). The transwell insert was placed in a lower chamber filled with 600 μL of serum-containing medium (10% FBS). Cell migration was performed for 24 or 48 h of incubation at 37 °C, and the transwell inserts were fixed and wiped out the upper side unmigrated cells. The inserts were stained with 0.1 mg/mL of crystal-violet, and the crystal-violet-stained cells were quantitatively analyzed according to the study of Luo et al. [56]. Briefly, the crystal violet-stained cells were dissolved in 250 μL of 20% acetic acid and the absorbance (O.D. 595 nm) was measured using an ELISA reader (Varioskan LUX Multi-mode Microplate Reader, Thermo Fisher Scientific, Waltham, MA, USA). The plots of proliferation were analyzed using the GraphPad Prism 8 Software (GraphPad, San Diego, CA, USA). For the detachment assay, at the indicated time points in the figure legends, the cell media from the lower chamber were removed, and the cells were fixed in 70% ethanol and washed twice with phosphate-buffered saline (PBS). Cells that had migrated to the bottom of the lower chamber were counted under a microscope (at 100× magnification). The number of cells at the bottom was counted from eight random fields in each experiment, and each assay was repeated in three independent experiments. Migration and detachment were analyzed using GraphPad Prism 8 (GraphPad, San Diego, CA, USA).

### 4.4. Three-Dimensional In-Gel Spheroid Detachment Assay

BFTC909 cells were used for 3D sphere formation, as previously described with modification [57]. Briefly, for 3D sphere formation, 1 × 10^4^ cells were resuspended in 100 μL of complete medium and centrifuged at 1200 rpm for 5 min. The aggregated cells were cultured at 37 °C in an incubator with a humidified atmosphere of 5% CO_2_. After 5 days, the wells of a 96-well plate were pre-coated with Matrigel (1:1 dilution) for 30 min at 37 °C, and the spheres were mixed with Matrigel (1:6 dilution) and then seeded on top of the pre-coated Matrigel. According to the desired experiment, medium with or without serum was added overnight. The medium was replaced with fresh medium plus 10% FBS, and the dissociated cells from 3D spheres were counted and measured using Image J (version 1.8, NIH, Bethesda, MD, USA).

### 4.5. Wound Healing Assay

Cells were counted (1–2 × 10^4^ cells/well) and then seeded in a 2-well culture insert (ibidi, Martinsried, Germany) with 100 μL of complete medium and left to settle overnight at 37 °C in an incubator with a humidified atmosphere of 5% CO_2_. After 4 h, an additional 20 μL of medium was supplied to avoid aggregation. The culture inserts were removed when 95% confluent and the medium was replaced with serum (10% FBS). Cell migration was performed for 24 or 48 h of incubation at 37 °C with the addition of desired chemicals or antibodies. Cells were fixed with 4% paraformaldehyde for 30 min, followed by immunofluorescence staining. In experiments involving actin cytoskeleton regulation, cells were incubated with latrunculin B or TGF-β1-blocking antibody for the desired time, as indicated in the figure legend.

### 4.6. Immunofluorescence Assay

Cells were fixed with 4% paraformaldehyde for 30 min and washed with PBS three times. Fixed cells were permeabilized with 0.1% Triton X-100 in PBS for 20 min and washed with PBS. The cells were then blocked in 1% BSA plus 0.1 M of glycine for another 30 min and washed with PBS-T (0.1% Tween-20). The cells were incubated with the indicated primary antibodies at 4 °C overnight. The next day, the cells were washed with PBS-T and incubated with secondary antibodies conjugated with Alexa Fluor 488 for 2 h. The washed cells were mounted using ProLong Gold Antifade Mountant with DAPI (Thermo Fisher Scientific, Waltham, MA, USA). In experiments involving actin cytoskeleton regulation, cells were incubated with latrunculin B or TGF-β1-blocking antibody for the desired time, as indicated in the figure legend. Images were taken using an Fv10i confocal microscope (Olympus, Tokyo, Japan) with a Plan Apochromat N 60×/1.40 silicon oil objective with z stacks. The intensity of fluorescence was analyzed with the Image J software (version 1.8, NIH, Bethesda, MD, USA). To determine the distance between vimentin and the cell membrane, the longest distance from the vimentin filament to the cell perimeter was measured. Eight field images were analyzed per sample using ImageJ software (version 1.8).

### 4.7. ELISA of Secreted TGF-β1

The protocol used to detect secreted TGF-β1 was performed according to the manufacturer’s instructions. The ELISA assay was used to detect TGF-β1 levels in a 96-well black plate format. For the detection of secreted TGF-β1, the Boyden chamber transwell assay was performed as described previously. Cancer cells (2 × 10^4^ cells/well) were seeded in the upper chambers overnight in complete medium. On day 2, the transwell insert was placed in a lower chamber with or without latrunculin B, and TGF-β1-blocking antibody was added into the medium of the lower chamber as desired in some experiments. TGF-β1 levels in the medium from the lower chamber were determined using the human TGF-β1 ELISA kit (ab100647, Abcam, Cambridge, MA, USA).

### 4.8. Statistical Analysis

Unless otherwise stated, all in vitro experiments were conducted in at least three separate experiments. Data from the 2.5D/3D detachment assay, immunofluorescence assay, and ELISA assay in this study are expressed as mean ± s.d. Statistical significance between different experimental groups was analyzed using the Student’s *t*-test (two-tailed), one-way ANOVA with Dunnett’s multiple comparisons test, and two-way ANOVA with Tukey’s multiple comparisons test. Statistical significance was set at *p* < 0.05. Statistical analyses were performed using GraphPad Prism 8 (GraphPad, San Diego, CA, USA).

## 5. Conclusions

TGF-β is known to have both positive and negative roles in tumor development, but the mechanism underlying its role in maintaining the balance of cancer cell adhesion and tumor detachment remains unclear. We established the Boyden chamber and 3D in-gel spheroid detachment assays and found that, under high cell density and adequate nutrients, TGF-β blockage contributed to the unbalance. Using a TGF-β-blocking antibody or actin polymerization inhibitor increased cancer cell dissociation from the transmembrane or tumor surface. Vimentin participated in TGF-β-regulated cytoskeletal remodeling, and the silencing of vimentin can obviously cause the invasive leading cell dissociation. TGF-β signaling mediated tumor invasiveness and enhanced TGF-β signaling induced cancer cell anchoring, whereas weakened TGF-β signaling was involved in the detachment in tumors with adequate nutrients.

## Data Availability

Data may be obtained from the corresponding author on request.

## Supplementary Materials

The following are available online at https://www.mdpi.com/article/10.3390/cancers13225648/s1. Figure S1: Effect of TGF-β1 blocking antibody on proliferation in the Boyden chamber detachment assay. Figure S2: Effect of TGF- β1 blocking antibody and latrunculin B on migration in the wound healing assay. Figure S3: Effect of mitomycin C on cell dissociation in the Boyden chamber detachment assay.
