# Supplementary material for: Dysregulation of Cytoskeleton Remodeling Drives Invasive Leading Cells Detachment
Source: Cancers (Basel). 2021 Nov 11;13(22):5648. doi: 10.3390/cancers13225648 (PMC8616115; doi:10.3390/cancers13225648)
Supplement: Supplementary file 1 [file cancers-13-05648-s001.zip › cancers-1409052-supplementary.pdf]

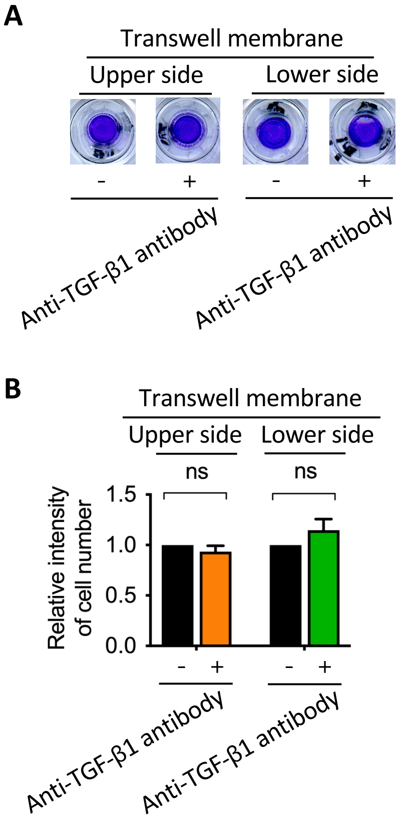

Supplementary Figure S1. Effect of TGF-β1 blocking antibody on proliferation in the Boyden chamber detachment assay. (A) Images of crystal violet staining from the representative upper chambers. BFTC909 cells were seeded in upper chamber overnight, the detachment assay was performed for 48 h with serum in the upper chamber and TGF-β1 blocking antibody in the lower chamber. (B) The crystal violet assay was performed and the relative cell number from the membranes of upper chamber was calculated. **, $p < 0.01$. Data are means ± s.d. (two-tailed t-test). LatB, latrunculin B.

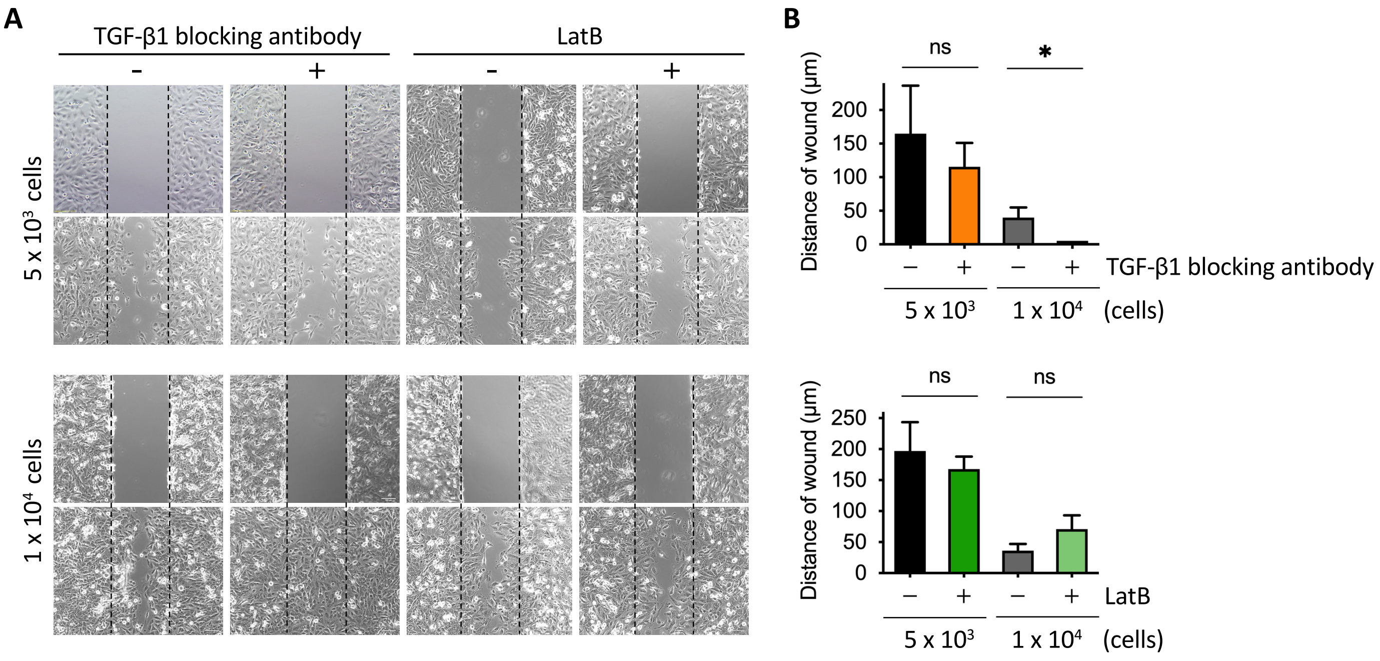

Supplementary Figure S2. Effect of TGF- β1 blocking antibody and latrunculin B on migration in the wound healing assay. (A) Images from the representative wound healing assay. BFTC909 cells were seeded in wound healing chamber overnight and migration was performed for 18 h with or without TGF-β1 blocking antibody or latrunculin B (0.5 μM). (B) Distance of wound was measured. Scale bars: 100 μm. *, $p < 0.05$. Data are means ± s.d. (two-tailed t-test). LatB, latrunculin B.

A

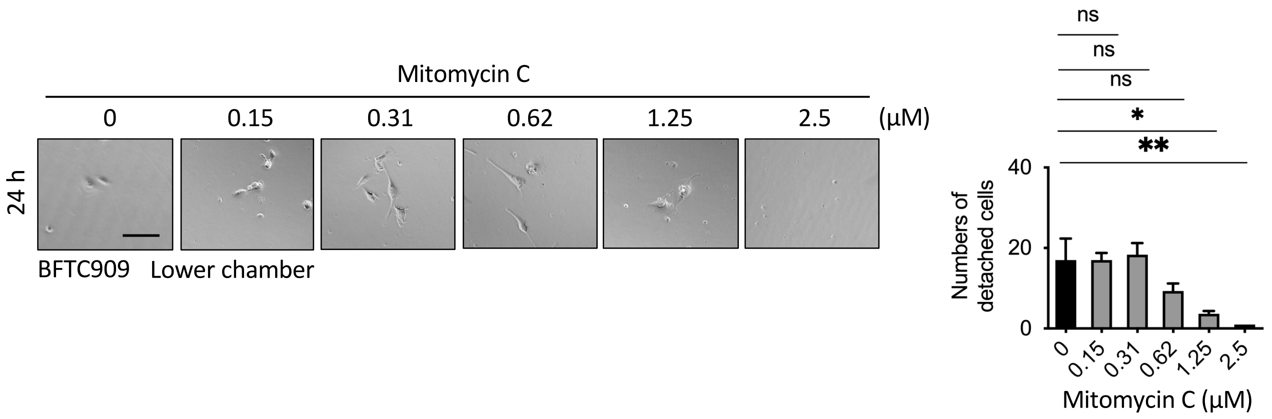

Supplementary Figure S3. Effect of mitomycin C on cell dissociation in the Boyden chamber detachment assay. (A) Images from the representative Boyden chamber detachment assay. BFTC909 cells were seeded in upper chamber overnight, the detachment assay was performed for 24 h with serum in the upper chamber and mitomycin C in the lower chamber. The cell number from lower chamber was measured. Scale bars: 100 μm. *, $p < 0.05$; **, $p < 0.01$. Data are means ± s.d. (two-tailed t-test).
